# Identification of major QTLs underlying tomato spotted wilt virus resistance in peanut cultivar Florida-EP^TM^ ‘113’

**DOI:** 10.1186/s12863-016-0435-9

**Published:** 2016-09-06

**Authors:** Yu-Chien Tseng, Barry L. Tillman, Ze Peng, Jianping Wang

**Affiliations:** 1 Agronomy Department, University of Florida, 2033 Mowry Road, Room 337 Cancer/Genetics Research Complex, Gainesville, FL 32610 USA; 2 North Florida Research and Education Center, University of Florida, Marianna, FL 32446 USA; 3 Genetics Institute, Plant Molecular and Cellular Biology Program, University of Florida, Gainesville, FL 32610 USA

**Keywords:** *Arachis hypogea*, Peanut, Tomato spotted wilt virus, SSR, Quantitative trait loci (QTLs), Marker-assisted selection (MAS)

## Abstract

**Background:**

Spotted wilt caused by tomato spotted wilt virus (TSWV) is one of the major peanut (*Arachis hypogaea* L.) diseases in the southeastern United States. Occurrence, severity, and symptoms of spotted wilt disease are highly variable from season to season, making it difficult to efficiently evaluate breeding populations for resistance. Molecular markers linked to spotted wilt resistance could overcome this problem and allow selection of resistant lines regardless of environmental conditions. Florida-EP^TM^ ‘113’ is a spotted wilt resistant cultivar with a significantly lower infection frequency. However, the genetic basis is still unknown. The objective of this study is to map the major quantitative trait loci (QTLs) linked to spotted wilt resistance in Florida-EP^TM^ ‘113’.

**Results:**

Among 2,431 SSR markers located across the whole peanut genome screened between the two parental lines, 329 were polymorphic. Those polymorphic markers were used to further genotype a representative set of individuals in a segregating population. Only polymorphic markers on chromosome A01 showed co-segregation between genotype and phenotype. Genotyping by sequencing (GBS) of the representative set of individuals in the segregating population also depicted a strong association between several SNPs on chromosome A01 and the trait, indicating a major QTL on chromosome A01. Therefore marker density was enriched on the A01 chromosome. A linkage map with 23 makers on chromosome A01 was constructed, showing collinearity with the physical map. Combined with phenotypic data, a major QTL flanked by marker AHGS4584 and GM672 was identified on chromosome A01, with up to 22.7 % PVE and 9.0 LOD value.

**Conclusion:**

A major QTL controlling the spotted wilt resistance in Florida-EP^TM^ ‘113’ was identified. The resistance is most likely contributed by PI 576638, a *hirsuta* botanical-type line, introduced from Mexico with spotted wilt resistance. The flanking markers of this QTL can be used for further fine mapping and marker assisted selection in peanut breeding programs.

**Electronic supplementary material:**

The online version of this article (doi:10.1186/s12863-016-0435-9) contains supplementary material, which is available to authorized users.

## Background

Cultivated Peanut (*Arachis hypogaea* L, 2n = 4x = 40) is a member of the legume family and is cultivated mainly in semi-arid tropic and sub-tropic regions. Worldwide, more than 42 million tons of peanuts were produced in 2013. Peanut seeds have high concentrations of oil (45–52 %) and protein (about 25 %), serving as a principal nutrition source in some developing countries [1]. Cultivated peanut is an allotetraploid with a genome composition of AABB. The genomes A and B were most likely derived from two wild diploid *Arachis* species, *A. duranensis* (A genome, 2n = 20) and *A. ipaensis* (B genome, 2n = 20) respectively [2]. Peanut production is significantly affected by spotted wilt disease caused by *Tomato spotted wilt virus* (TSWV) (genus *Tospovirus*, family *Bunyaviridae*), specifically in the United States. In 1997, the production losses due to spotted wilt in Georgia were estimated to be approximately $40 million [3]. The typical symptoms of spotted wilt on peanuts are yellowing, stunting, concentric ringspots, chlorosis, and necrosis of various sizes and shapes on leaflets [4].

Many factors affect the severity of spotted wilt including peanut variety, planting date, plant population, row pattern, crop rotation, and tillage. Host resistance (peanut variety) is the most important factor to reduce disease risk. Hence, the development of spotted wilt resistance varieties has become a major breeding objective in peanut breeding programs in the United States. Traditionally, peanut breeders select resistant plants in the field under natural conditions. However, expression of spotted wilt disease is highly variable from season to season, reducing selection efficiency. The recent advances in genetic and genomic tools and resources for peanut increased the potential of using molecular markers for selection to accelerate the peanut cultivar improvement [5]. Much progress was made in the past few years. Specifically, the two ancestor genomes, the A genome from *A. duranensis* and the B genome from *A. ipaensis*, have been sequenced and annotated [6], which provided a fundamental resource for molecular marker development.

Simple sequence repeat (SSR) marker is a valuable type of marker. Its abundance in the genome, co-dominance, multiple alleles, high polymorphism, PCR-based simple analysis, and transferability from other related species made SSR marker a great choice [7, 8] for peanut genetic studies. Single nucleotide polymorphism (SNP) is another type of molecular marker, which is very abundant and distributed throughout the whole genome. Those molecular markers can be used to help construct peanut linkage maps [9–11]. Linkage maps provide the basic framework for genetic and genomics studies, such as quantitative trait locus (QTL) analysis, marker assisted selection (MAS), comparative genomics, and genome assembly. QTL analysis based on the linkage map is critical in identifying markers linked to agronomically important traits. Several QTLs for important traits have been identified in peanut, such as drought tolerance, disease resistance, and nutritional quality [9, 12–15].

Florida-EP^TM^ ‘113’ is a new runner-type variety with superior spotted wilt resistance [16]. It has been tested under earlier planting date (April) and reduced seed density (13.1 seed per meter). Both conditions favor for spotted wilt epidemics. However, Florida-EP^TM^ ‘113’proved to have resistance sufficient to obviate high risk situations presented by earlier planting date and lower seed density [17]. One of the parental lines of Florida-EP^TM^ ‘113’ is NC94022. High level of field resistance has been reported for NC94022 [18] and the crosses utilizing NC94022 as a parental line in peanut breeding programs were initiated [19]. In 2012, Qin et al. [20] reported the first genetic linkage map based on NC94022-derived population and a major QTL asssociated with resistance to TSWV on linkage group ‘A1’ was reported, which had a phenotypic variation explained (PVE) of 35.8 %. Recently Khera et al. [21] reported an improved genetic map for the same NC94022-derived population and QTLs on chromosome ‘A01’ associated with multi-year TSWV phenotypic data were identified. However, whether genetic basis of the spotted wilt resistance in Florida-EP^TM^ ‘113’ is the same as the NC94022 was unknown. The objective of this study was to understand the genetic basis of the supurior spotted wilt resistance in Florida-EP^TM^ ‘113’ through maping and QTL analysis using Florida-EP^TM^ ‘113’-derived populations. If the major QTLs of the resistance in Florida-EP^TM^-‘113’ can be detected, MAS can be applied in the breeding program to avoid uncertain environmental impacts on selection of new cultivars.

## Methods

### Plant materials

An F_2_ population was derived from cross between Florida-EP^TM^ ‘113’ and Georgia Valencia made in 2009. Georgia Valencia is a large-podded Valencia market type peanut susceptible to spotted wilt [22]. The F_2_ segregating population comprised of 200 lines was planted at the North Florida Research and Education Center (NFREC) near Marianna, FL, in 2011. All F_2_ plants were self-pollinated to generate next generation seeds. Since seeds harvested from a few F_2_ lines were mixed up during seeds processing and thus discarded, only 163 pure F_2:3_ (F_2 -_derived in F_3_) families were kept and planted in 2012. Planting of F_2:3_ lines took place at Plant Science Research and Education Unit (PSREU) located near Citra, Florida in early April and at NFREC in the middle of April 2012. An augmented experimental design with two parental lines as controls was used at each location. Each plot had two rows, 0.9 m wide and 4.5 m long, and a single family was planted in each plot. Seed planting density was one seed per 0.3 m.

The plants from each F_2:3_ family allowed self-pollinating. The F_2:3_ seeds were bulk harvested and seeds from each family were randomly chosen to generate 163 F_2:4_ (F_2 -_derived in F_4_) families in 2013. After the harvest of the F_2:4_ families, 163 F_2:5_ (F_2 -_derived in F_5_) families were planted subsequently in 2014. The experimental design for the F_2:5_ was the same as the F_2:3_ and F_2:4_ but the planting took place only at the NFREC location (Additional file 1). A single plant was harvested from each F_2:5_ families and seeds were randomly chosen to plant the F_6_ generation as RILs (163 lines) with a high level of homozygosity. F_2:3_, F_2:4_ and F_2:5_ generations were included in the research for phenotyping.

### Rating for disease resistance

Two different disease evaluation methods were conducted to assess the severity of spotted wilt. One was a visual rating on a scale ranging from 1 to 10, and the other was a form of immunoassay (immunostrip testing), which was used to test for the presence of TSWV in the root crown. A visual rating was conducted on a whole plot basis prior to digging. Each plot was assessed for typical symptoms of spotted wilt such as stunting and foliar symptoms of ringspot, leaf necrosis, and chlorosis (yellowing) [4]. The 1 to 10 scale represented a percentage of infected plants (1, 1.5, 2, 3, 4, 5, 6, 7, 8, 9, 10 equals 0 %, 1–10 %, 11–20 %, 21–30 %, 31–40 %, 41–50 %, 51–60 %, 61–70 %71–80 %, 81–90 %, and 91–100 %, respectively).

The immunostrip testing was conducted by using the ImmunoStrip Kit (Agdia Inc., Elkhart, IN, USA). Ten individual plants were randomly harvested from each plot and root crowns of each plant were collected and air-dried after digging. Approximately 0.4 g root crown sample was trimmed and subjected to immunostrip test following the manufacturer’s instructions. The percentage of infected plants out of the 10 randomly selected plants from each plot represented the TSWV infection frequency of each plot, thus each plot had the possibility of TSWV infection percentage ranging from 0 to 100 %.

The visual disease evaluation method was conducted in F_2:3_ and F_2:4_ families at both NFREC and PSREU. For F_2:5_ families, only at NFREC, the evaluation was applied. Immunostrip testing was utilized in F_2:3_ and F_2:4_ generations at only NFREC (Additional file 1). Phenotypic correlations were analyzed using SPSS 22.0 to calculate Spearman’s rank correlation coefficient (Spearman’s rho).

### SSR genotyping

The genomic DNA of each F_2_ individual was extracted from approximately 500 mg young leaf tissues using the method described by Dellaporta et al. [23], with a modification that 1 % polyvinylpyrrolidone (PVP) was added to extraction buffer to remove phenolic compounds. DNA quality and quantity were evaluated by using 1 % agarose gel electrophoresis and NanoDrop (Thermo Scientific, United States). The isolated DNA was diluted to 5 to 30 ng/ul for further polymerase chain reaction (PCR) process.

Publicly available SSR markers were selected based on polymorphism information content (PIC) and linkage group information from related literatures [7–9, 11, 13, 14, 20, 24–39] (Additional file 2). The primers of selected SSR markers were synthesized by Invitrogen^TM^, Life Technologies. PCR was performed in 10 ul volumes containing 1 μl of 10 × PCR buffer, 1 μl of Magnesium Chloride (25 mM), 1 μl of dNTP (2 mM), 0.3 μl of Taq enzyme (home-made), 2 μl of forward and reverse primers (2 mM), 2 μl of DNA template (10 ng/μl), and 2.8 μl of distilled deionized water. The PCR program was operated using a touchdown program with an initial denaturation at 94 °C for 3 min; 10 cycles of amplification at 94 °C for 30 s, 65 to 55 °C for 20 s (every cycle drops one degree until 55 °C), 72 °C for 40 s; 30 cycles of amplification at 94 °C for 30 s, 55 °C for 20 s, 72 °C for 40 s; and a final extension at 72 °C for 7 min. The PCR products were separated on 6 % non-denatured polyacrylamide gel electrophoresis (PAGE) under 150 volts for 2 h in 1X TBE buffer with DYCZ- 30B gel rigs system (Beijing, China) [40]. The gels were stained by ethidium bromide and visualized under UV light.

### Genotyping by sequencing (GBS)

Two parental lines (Florida-EP^TM^ ‘113’ and Georgia Valencia) plus 10 F_6_ lines samples were selected for GBS. Among the 10 F_6_ lines, five lines consistently showed disease resistance for 3 years and the other five lines showed susceptibility. The 12 DNA samples with high quality and a concentration higher than 50 ng/ul were delivered to Cornell University, Institute of biotechnology (Genomic Diversity Facility) for 96-plex GBS library preparation (in combination with other 83 samples unrelated with this experiment) and sequencing with Illumina HiSeq 2000 platform following the optimized protocol [41]. The restriction enzyme, *Ape*KI was used for reduced representation library preparation.

The Tassel-GBS pipeline was utilized for SNP calling [42]. Trimmed and cleaned sequence tags were aligned to peanut A and B genomes respectively [6] using Bowtie2 [43]. The minor allele frequency (MAF) cutoff was set at 0.01. Raw SNPs were called and the resulting HapMap.hmp.txt files were input into TASSEL software [44] for further filtering. SNPs with more than 25 % missing data were removed. The remaining SNPs were applied for single marker analysis first. Then association mapping analysis was conducted. The general linear model (GLM) was performed and a Manhattan plot was generated by Tassel v5.0 after inputting phenotyping data of 12 samples [44, 45].

### Linkage and QTL analysis

Linkage analysis was performed using software QTL IciMapping V4.0 [46] in combination with JoinMap 4.0 [47]. To construct linkage groups, a minimum log-of-odds (LOD) threshold of 3.0 was applied and map distances were converted to centi morgans (cMs) using Kosambi mapping function [48]. The 3-year phenotyping data were incorporated with linkage map information for QTL mapping using the software QTL IciMapping V4.0 [46]. The Inclusive Composite Interval Mapping (ICIM-ADD) method [49, 50] was applied for QTL analysis with a minimum 3.0 LOD, 0.001 probability in stepwise regression, and a scanning interval of 1.0 cM/step.

## Results

### Disease rating distribution in the segregating populations

**Fig. 1:**
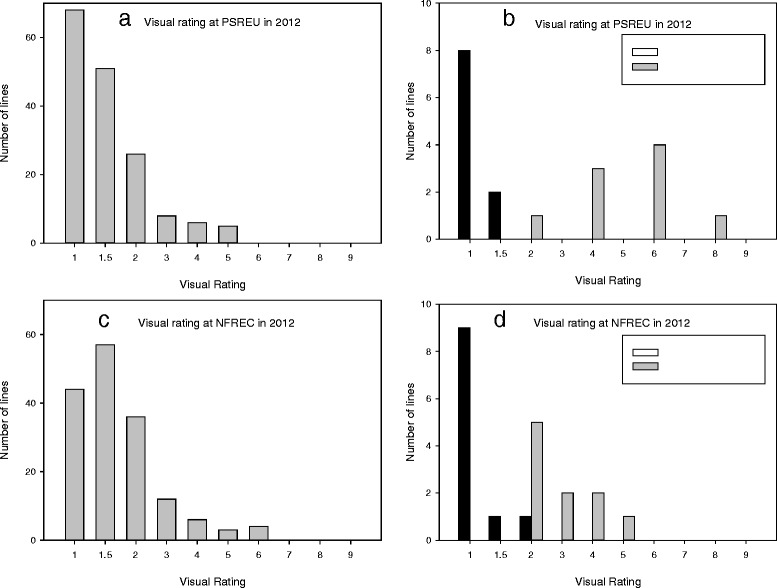
TSWV infection results on F_2:3_ population and parental lines (checks) by visual rating in 2012. **a** F_2:3_ population at PSREU; (**b**) checks of F_2:3_ population at PSREU; (**c**) F_2:3_ population at NFREC; (**d**) checks of F_2:3_ population at NFREC

Out of 163 F_2:3_ lines, more than 68 (40 %) at the PSREU in 2012 had a disease rating of “1”, indicating no visible symptoms. Only 25 lines showed more than 20 % disease symptoms (disease rating more than 2) (Fig. 1a). The two checks (two parental lines) showed a distinct difference at PSREU in 2012. Out of the 10 Florida-EP^TM^ ‘113’ plots, eight did not show symptoms, but all Georgia Valencia plots showed various levels of disease symptoms with ratings ranging from 2 to 8 (Fig. 1b). Compared to the rating data collected from PSREU, data from NFREC had more plots with ratings of 1.5 and less plots with ratings of 1 (Fig. 1c). Florida-EP^TM^ ‘113’ checks had mostly ratings of 1, while Georgia Valencia had a wide rating range (Fig. 1d). In general, the spotted wilt epidemics detected by visual rating was higher at NFREC than at PSREU.

**Fig. 2:**
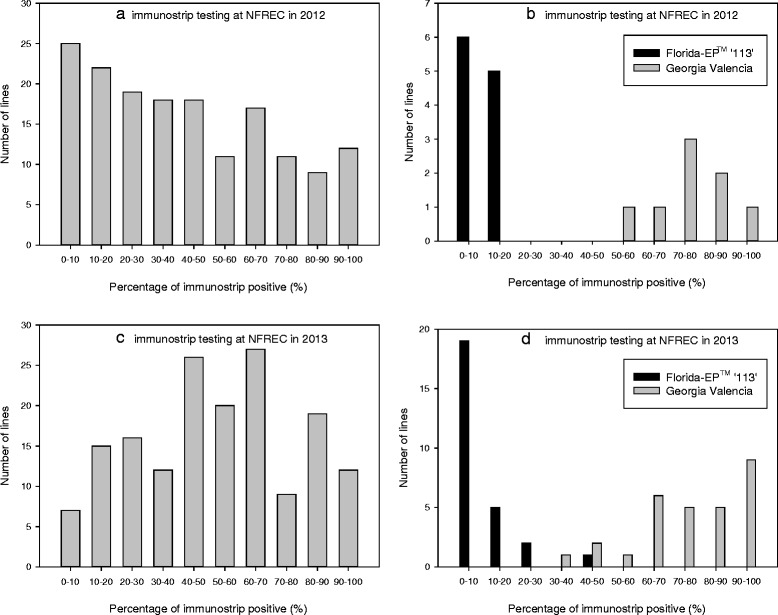
TSWV infection results on populations and parental lines (checks) by immuostrip testing in 2012 and 2013 at NFREC. **a** F_2:3_ population in 2012; (**b**) checks of F_2:3_ population in 2012; (**c**) F_2:4_ population in 2013; (**d**) checks of F_2:4_ population in 2013

The distribution of Immunostrip test results in 2012 was relatively even in contrast to the skewed distribution in visual ratings (Fig. 2a). The disease rating distributions of Florida-EP^TM^ ‘113” and Georgia Valencia were distinct in 2012 (Fig. 2b).

The distribution of visual ratings of the F_2:4_ population at PSREU in 2013 were skewed toward low disease rating. There were 132 plots, whose rating range were from 1, 1.5 to 2, 60 plots were scored 1.5; 34 plots were scored 1, and 38 plots were scored 2 (Additional file 3). Florida-EP^TM^ ‘113’ received all ratings below 2; however, Georgia Valencia also received many rating scores below 2 (Additional file 3). At NFREC, the visual rating resulted in most plots being scored 2 (45 plots), followed by rating score of 3 (38 plots). Only one plot was free of visual symptoms, whereas more plots had a high rating score (>3) (Additional file 3). Georgia Valencia plots had a wide range of disease ratings ranging from 1 to 9 indicating the uneven infection or disease development in the field (Additional file 3). In general, the disease pressure was higher in 2013 than in 2012, and the disease rating was higher at NFREC than at PSREU.

The distribution of immunostrip test results of the population in 2013 was nearly a normal distribution with a majority of plots falling between 40 and 70 % (Fig. 2c). As for the disease severity distribution of the two checks, it was bimodal, which represented their resistant and susceptible feature respectively (Fig. 2d).

The F_2:5_ population was only rated visually at NFREC, 2014. More lines (38, 35, and 33 lines, respectively) received rating scores of 2, 3 and 4 than other lower or higher rating scores (Additional file 4). Most plots of Florida-EP^TM^ ‘113’ received rating scores of 2 and lower, and the rating scores of Georgia Valencia plots were all higher than 2 (Additional file 4).

### Phenotypic correlation

A total of seven phenotypic datasets representing different years, locations, generations of the population, and measurement methods were recorded. The seven datasets were entitled as 2012NF-VR (2012: year 2012; NF: NFREC; VR: visual rating), 2013NF-VR (2013: year 2013; NF: NFREC; VR: visual rating), 2014NF-VR (2014: year 2014; NF: NFREC; VR: visual rating), 2012PS-VR (2012: year 2012; PS: PSREU VR: visual rating), 2013PS-VR (2013: year 2013; PS: PSREU; VR: visual rating), 2012NF-IS (2012: year 2012; NF: NFREC; IS: immunostrip), and 2013NF-IS (2013: year 2013; NF: NFREC; IS: immunostrip). There were a total of 21 possible pair-wise combinations among the seven datasets. The correlations of all the combinations were significant, except the combination between 2012NF-VR and 2013PS-VR (Table 1).

**Table 1:** Spearman’s rank correlation coefficients among the phenotypic measures of spotted wilt in peanut tested in Marianna and Citra, FL

	2012NF-VR	2012PS-VR	2013NF-IS	2013NF-VR	2013PS-VR	2014NF-VR
2012NF-IS	0.591**	0.270**	0.498**	0.503**	0.209**	0.290**
2012NF-VR		0.266**	0.285**	0.355**	0.094	0.162*
2012PS-VR			0.247**	0.266**	0.236**	0.223**
2013NF-IS				0.781**	0.401**	0.433**
2013NF-VR					0.316**	0.343**
2013PS-VR						0.338**

* means correlation is significant at the 0.05 level, ** means correlation is significant at the 0.01 level. NF = North Florida Research and Education Center, Marianna, FL; PS = Plant Science Research and Education Unit, Citra, FL. IS = Immunostrip Testing; VR = Visual Rating

The highest correlation was found between 2013NF-IS and 2013NF-VR with a coefficient of 0.78 (*p* < 0.01). The second highest correlation coefficient was 0.59 (*p* < 0.01) between 2012NF-IS and 2012NF-VR. In general, the datasets from the same location tended to have higher correlations than those from different locations, indicating environmental variation between the two locations. Even though 2012NF-IS and 2013NF-IS were datasets of different years and generations, they were still correlated with a coefficient of 0.498 (*p* < 0.01). However, 2012NF-IS and 2013NF-VR were also correlated with a coefficient of 0.503 (*p* < 0.01), but they were datasets from different years and using different measurement methods. Overall, the majority of the correlations had coefficients in a range between 0.2 and 0.4. While comparing the correlations within the year of 2013 (0.781, 0.401, and 0.316, *p* < 0.01) and within the year of 2012 (0.591, 0.270, and 0.266, *p* < 0.01), the correlations of datasets collected in 2013 were higher than those in 2012.

Within the correlations of datasets between 2012 and 2013 that used the same measurement method (visual rating), the dataset generated from NFREC had greater correlation coefficient (0.355, *p* < 0.01) than that from PSREU (0.236, *p* < 0.01). While comparing the correlations between datasets in 2012 and 2013 at the same location (NFREC), the correlation coefficient of the IS (0.498, *p* < 0.01) was higher than that of the VR (0.355, *p* < 0.01).

### SSR marker screening

A total of 2,431 markers across the whole peanut genomes (Table 2) were screened between the two parental lines, Florida-EP^TM^ ‘113’ and Georgia Valencia. Each linkage group (LG) had an average of 88.4 markers screened, ranging from 62 to 128 markers/LG. The average number of markers screened was 93.9 on each A chromosome and 82.9 on each B chromosome, with additional 663 markers having no linkage group information. The sequences from 2,431 markers were aligned to A and B reference genomes [6] and 1,637 (67.34 %) could be mapped to the genomes and 794 SSR primers (32.66 %) could not be mapped, which may due to the genome rearrangement of cultivated peanut or incompleteness of the reference genomes.

**Table 2:** The number of SSR primers screened, amplifible and polymorphic according to different linkage groups in cross between Florida-EP^TM^ ‘113’ and Georgia Valencia peanut cultivars

Linkage Group	Total SSRs Screened	Number of Amplifiable Primer	Number of Polymorphic Primer	Amplification Ratio (%)	Polymorphic Ratio (%)
A01	110	98	19	89.09	17.27
A02	75	66	14	88.00	18.67
A03	128	121	23	94.53	17.97
A04	100	88	7	88.00	7.00
A05	89	81	9	91.01	10.11
A06	98	92	14	93.88	14.29
A07	62	54	15	87.10	24.19
A08	102	93	3	91.18	2.94
A09	91	82	20	90.11	21.98
A10	84	77	6	91.67	7.14
B01	99	89	20	89.90	20.20
B02	72	64	19	88.89	26.39
B03	95	89	8	93.68	8.42
B04	97	90	7	92.78	7.22
B05	76	72	8	94.74	10.53
B06	82	78	10	95.12	12.20
B07	80	72	13	90.00	16.25
B08	82	75	6	91.46	7.32
B09	75	66	15	88.00	20.00
B10	71	69	11	97.18	15.49
No linkage group information	663	606	82	91.40	12.37
Total	2431	2221	329	91.36	13.53

The number of amplifiable markers was 2,221 with an amplification ratio of 91.36 %. Polymorphism was detected between the two parental lines at 329 SSR marker loci with a polymorphic ratio of 13.51 %. The lowest and highest polymorphic ratio was found on A04 (2.94 %) and B02 (26.39 %), respectively (Table 2).

### Major QTL identification

**Fig. 3:**
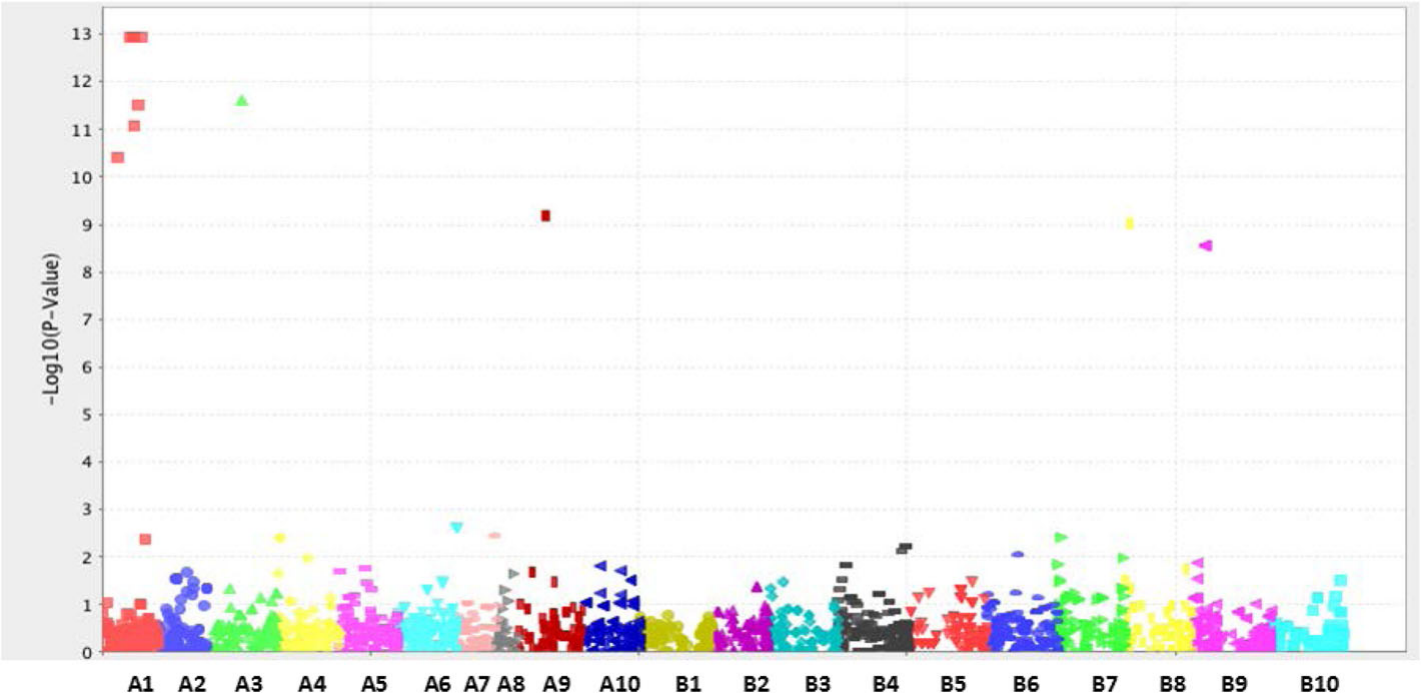
Manhattan plot showing the associations between SNPs and spotted wilt resistance on different peanut chromosomes. Different colors indicated different chromosomes on X-axis and every single data points represented each SNP. Y-axis,indicated the negative logarithm of the association *P*-value. The higher numbers mean stronger associations between traits and SNPs

To identify the chromosome location of the major QTLs for TSWV resistance, the 329 polymorphic SSR markers were further screened using 12 selected individuals, consisting of two parental lines, five susceptible and five resistant lines. Only 19 markers on chromosome A01 displayed co-segregation with phenotypes (Additional file 5). Markers on the other 19 chromosomes showed random band patterns and did not correspond to their phenotypic data (Additional file 5) indicating that potential major QTLs of spotted wilt disease resistance in Florida-EP^TM^ ‘113’were on the chromosome A01. In addition, the GBS experiment generated a total of 1,856,429 sequences reads and 7,972 raw SNPs were called by Tassel-GBS pipeline. After removing the SNPs with high level of missing data (>25 %), a total of 2,670 SNPs were identified. Single marker analysis indicated that 18 SNP markers co-segregated with phenotypes of the 12 individuals including 8 SNPs on A01 chromosome, two on B7 chromosome one marker on A3, A4, A6, A7, A8, A9, B8 and B9 chromosome respectively. Association mapping further confirmed some of the markers associated with the TSWV resistance as showed on the manhattan plot (Fig. 3). The results further indicated that there were major loci on chromosome A01 associated with spotted wilt disease resistance (Fig. 3). Chromosomes A3, A9, B8 and B9 also showed a few SNPs associated with TSWV resistance. However, compared to the number of associated SNPs on chromosome A01, the numbers of SNPs on other chromosomes were relatively few.

To increase the mapping resolution on chromosome A01, an additional 154 SSR markers from latest literature [9, 11, 34] on chromosome A01 were utilized for polymorphism screening using the parental lines. Overall, 2,583 markers have been screened (Additional file 2) and 29 polymorphic SSR markers on A01 were used to genotype the whole F_2_ population of 163 individuals.

**Fig. 4:**
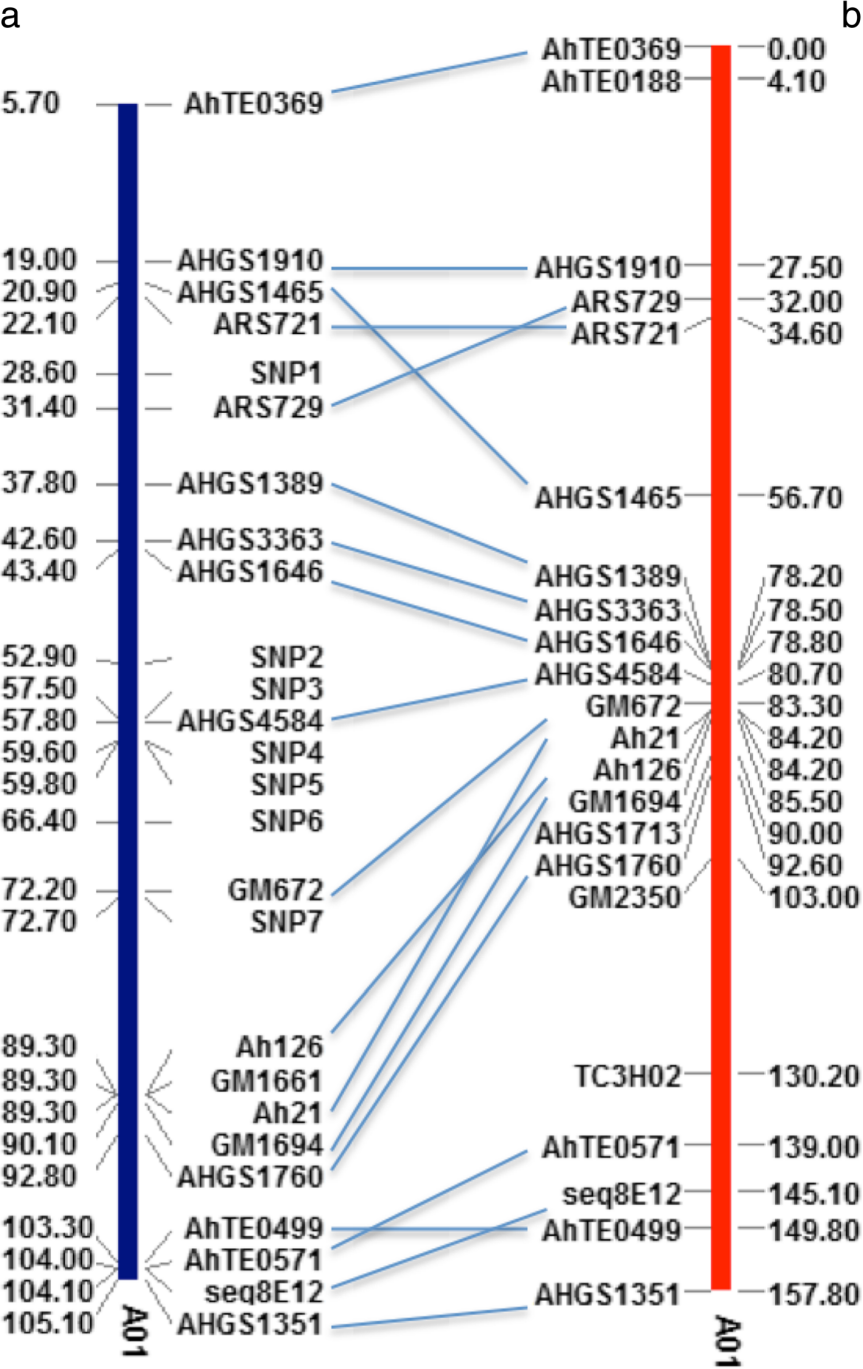
Physical and linkage map showing the position of SSR and SNP markers on chromosome A01. Lines indicated the same markers on both maps. **a**) physical map with the numbers indicating Megabase (Mb); **b**) linkage map with numbers indicating Centimorgan (cM)

Linkage analysis revealed that out of 29 polymorphic SSR markers 23 markers were mapped on one linkage group. The genetic distance was 157.80 cM (Fig. 4b). Out of 23 linked SSR markers, 19 markers can be aligned to the reference genome on chromosome A01. The physical positions of seven SNP markers from GBS were also indicated on the physical map (Fig. 4a). On the physical map, the top marker aligned to A01 was AhTE0369 and the position was at 5.7 megabase. The bottom marker was AHGS1351 and the position was at 105.1 megabase. A high collinearity between the genetic and physical maps was observed (Fig. 4, Table 3).

**Table 3:** The positions of A01 markers on physical (Mb) and linkage map (cM) in cross between Florida-EP^TM^ ‘113’ and Georgia Valencia peanut cultivars

Marker name	Physaical map (Mb)	Linkage map (cM)
AhTE0369	5.692568	0
AhTE0188	-	4.11
AHGS1910	19.035441	27.49
SNP1	28.63	-
ARS729	31.451985	32.03
ARS721	22.130732	34.56
AHGS1465	20.917612	56.67
AHGS1389	37.76887	78.21
AHGS3363	42.634304	78.52
AHGS1646	43.349687	78.83
SNP2	52.87	-
SNP3	57.55	-
AHGS4584	57.79209	80.73
SNP4	59.65	-
SNP5	59.79	-
SNP6	66.38	-
GM672	72.196023	83.28
SNP7	72.67	-
Ah21	89.295856	84.22
GM1661	89.295751	82.22
Ah126	89.295748	84.22
GM1694	90.064266	85.46
AHGS1713	-	90
AHGS1760	92.841619	92.57
GM2350	-	103.02
TC3H02	-	130.17
AhTE0571	103.985083	139.01
seq8E12	104.139715	145.06
AhTE0499	103.336438	149.76
AHGS1351	105.122604	157.85

**Fig. 5:**
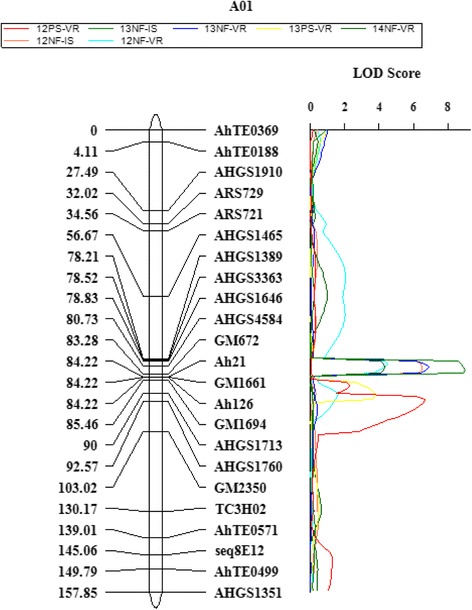
Linkage group with SSR marker positions and the detected QTLs indicated by different color peaks indicating different phenotyping datasets

The seven phenotypic datasets and the genotypic results were used for QTL analysis. Two QTLs representing two locations, NFREC and PSREU, were detected on chromosome A01 (Fig. 5). The same QTL was identified using phenotypic datasets of 2012NF-IS, 2013NF-VS, 2013NF-IS, and 2014NF-VS with flanking markers, AHGS4584 (80.73 cM) and GM672 (83.28 cM). A similar QTL was identified using 2012NF-VS dataset with flanking markers, AHGS1646 (78.83 cM) and AHGS4584 (80.73 cM). The QTL using 2013NF-IS dataset showed the highest LOD score (9.00) and PVE (22.7 %) (Table 4). QTL identified using datasets of 2012PS-VS and 2013PS-VS had same flanking markers, AHGS1713 (90.00 cM) and AHGS1760 (92.57 cM). However, the QTL identified using 2013PS-VS dataset showed the lowest LOD score (3.76) and PVE (10.02 %) (Table 4).

**Table 4:** The positions, flanking markers, LOD values, PVE (%) and additive effects of putative QTLs on A01 chromosome in cross between Florida-EP^TM^ ‘113’ and Georgia Valencia peanut cultivars

Dataset	Chromosome	Position (cM)	Left marker	Right marker	LOD	PVE (%)	add
2012PS-VR	A01	92	AHGS1713	AHGS1760	6.65	16.93	−0.56
2013PS-VR	A01	90	AHGS1713	AHGS1760	3.76	10.02	−0.24
2012NF-VR	A01	80	AHGS1646	AHGS4584	4.52	12.17	−0.59
2013NF-VR	A01	81	AHGS4584	GM672	6.91	17.69	−0.91
2014NF-VR	A01	81	AHGS4584	GM672	4.33	11.55	−0.71
2012NF-IS	A01	81	AHGS4584	GM672	6.52	17.06	−0.19
2013NF-IS	A01	82	AHGS4584	GM672	9	22.7	−0.17

## Discussion

### Phenotypic variability in population

Mechanical transmission of TSWV is difficult to achieve. Several factors affect the transmission efficiency [51]. In addition, it is time consuming to inoculate every single plant in large-scale breeding experiment. In this study, the phenotypic data were collected based on natural inoculation. Under natural inoculation conditions, single plant rating is not reliable for spotted wilt disease evaluation, so the plot-based evaluation with multiple years at two different locations was applied in this study. Each line in the F_2:4_ and F_2:3_ families can be traced back to a single F_2_ plant. The family mean of phenotypic value from multiple individuals can represent the phenotype of the single F_2_ plant [52].

Visual rating was a comprehensive plot-based method including the assessment of spotted wilt severity and incidence. However, compared to immunostrip test, visual rating had a narrow disease scoring scale (1 to 10). When conducting QTL analysis, the discrete scales might not be able to represent the diverse variability of the populations. In addition, visual rating relies on experience and thus is relative subjective and may produce rating biases by a single person.

Immunostrip results were derived from 10 random plants in a plot and can be considered as the average of a plot. Immunostrip testing can capture TSWV reaction more accurately than visual scoring, since it can detect the virus in non-symptomatic plants. Approximately 50 % of non-symptomatic plants of some cultivars were found to be infected with TSWV [17]. This means that visual rating does not capture the potential of disease expression. Visual rating resulted more ratings in the low range (rating 1, 1.5 and 2) and much fewer in the high range (rating 7, 8 and 9) mostly due to the non-symptomatic issue and non-synchronized infection. Varying disease pressure over seasons and locations can affect the spotted wilt epidemic, making visual ratings less reliable. Even the homozygous parental lines exhibited inconsistent disease levels across the field, specifically when disease pressure was not sufficient; the susceptible and resistant checks were not easily distinguished. However, the distribution of immunostrip testing results of the two parents showed a distinction. According to immunostrip results, more plots had severe infection compared to those with no or minor infection.

### Major QTL underlying the TSWV resistance in Florida-EP^TM^ ‘113’

Bulk segregant analysis based on polymorphic SSR markers indicated that only markers located on A01 chromosome showed co-segregation with resistant/susceptible plants (Additional file 5). In addition, according to association analysis based on GBS data (Fig. 5), 11 SNPs showed a high degree of association with spotted wilt resistance (−log_10_ (*p*-value) = 8 as cutoff) and 7 of 11 SNPs were located on chromosome A01. The other four were located at chromosome A3, A9, B8, and B9 respectively, most likely representing minor QTLs, which will be investigated in the future. In this study, we focused on the major QTLs located at chromosome A01.

The QTL analysis in this study (Table 4 and Fig. 5) revealed two putative QTLs on chromosome A01 next to each other. One was associated with NFREC phenotypic data and the other was associated with PSREU phenotypic data, which most likely are the same QTL with a slight shift to different positions due to the environment effects [53]. In addition, genotype-by-environment interaction, population size, marker density, and genotyping errors [54] can also influence the QTL position. Therefore, most likely one major QTL on chromosome A01 with 23 % PVE controls the spotted wilt disease resistance in Florida-EP^TM^ ‘113’. Association analysis based on GBS data revealed seven SNPs on chromosome A01 associated with TSWV resistance with three of them between flanking markers AHGS 4584 and GM 672. Two are between markers AHGS 1646 and AHGS4584 (Fig. 3). The resutls were in accordance with the QTL mapping (Fig. 4 and Table 3). In this study, the map was consructed based on a F_2_ popualtion, which might lead to overestimated phentoypic effects of QTL due to large number of heterozygous loci in the F_2_ individuals and transgressive segreation [55]. Further validation of the effects of this major QTL with advanced RIL population may be necessory.

Qin et al. [20] and Khera et al. [21] also reported major QTLs associated to TSWV resistance on chromosome A01. Due to large number of different polymorphic markers between the maps, we noticed that one (ARS721) of the two flanking markers (Seq12F7 and ARS721) of the QTL identified by Qin et al. [20] and two markers (Ah21 and Ah126) of the four flanking markers (Ah21, Ah126, Seq13A10 and GNB842) of QTLs identified by Khera et al. [21] were also mapped to A01 linkage group in our study. Based on the physical locations of these common SSR markers (Table 3), we could tell that our QTL location flanked by AHGS4584 and GM 672 were between the QTLs identified by Qin et al. [20] and Khera et al. [21], not quite the same as either of them. It could be either that the major QTL related to TSWV resistance in Florida-EP^TM^ ‘113’ was different from that in the NC94022 or most likely that these reported QTLs earlier were the same but with shifted location due to environment effects [53]. There was another possible TSWV resistance QTL located on chromosome A09 [21]. Five markers showed polymorphism between two parental lines on chromosome A09 were further used to genotype 163 F_2_ plants in our experiment, however, these markers were not linked together based on this mapping population. Our association analysis using GBS data indicated one SNP on chromosome A09 significantly associated with TSWV resistance, which might be a minor QTL and will be investigated in the future.

### Spotted wilt resistance QTLs

Spotted wilt dominant resistance genes have been found in tomato (*Solanum lycopersicum*) and pepper (*Capsicum annuum*), named *Sw-5* and *Tsw*, respectively with the hypersensitive response (HR) resistant mechanism [56, 57]. In this study, the spotted wilt resistance in Florida-EP^TM^ ‘113’ does not appear to be a HR mechanism, although one major QTL was identified. *Sw-5* has an ortholog gene in peanut, *Ahsw* with 37 % amino acid identity to *Sw-5* [58]. The gene was characterized as peanut oxalate oxidase [59] and two gene-specific SSR markers (Seq2F10 and TC7G10) were identified. However, none of them displayed polymorphism between Florida-EP^TM^ ‘113’ and Georgia Valencia, thus were not used to validate the linkage within our segregating population.

Two mapping populations have been utilized for spotted wilt resistance QTL mapping previously. They were derived from the cross between Tifrunner and GT-C20 (referred as T-population) and the cross between SunOleic 97R and NC94022 (referred as S-population) [20]. Two resistant QTLs (*qTSWV1* and *qTSWV2*) were first reported on S populations [20]. *qTSWV1* was located on linkage group 15 (LGJ15) on T-population and *qTSWV2* was on A01 chromosome on S-population. Further 15 and 9 QTLs were identified using different generations (F_2_ and F_5_) on T-population [55], respectively. With S-population, there were six spotted wilt related QTLs were identified [21]. The major QTL located on LG A01 identified based on S population was at the similar location as the QTLs identified in the study here. Most likely the resistance segregating in both populations was derived from same genetic source, PI 576638, known as *hirsuta* botanical-type line introduced from the highlands of Mexico.

Peanut is not a native crop to the United States, so plant introduction (PI) played an important role in peanut cultivar development. Two PIs, PI 203396 and PI 576638, have provided extensive disease resistant sources [60, 61]. It was reported that several breeding lines containing PI 576638 origin had better TSWV resistance than the lines derived from PI 203396. The two PI accessions may contain different resistant genes and have different resistance mechanisms [18]. At the similar QTL region on A01, QTL for early leaf spot resistance was also detected [21], indicating that wild *Arachis* species displayed a high level resistance to several diseases (early leaf spot, late leaf spot, and stem rot) [62].

### Marker assisted selection (MAS)

In this study, the spotted wilt resistant QTL with 22.7 % PVE was identified and the two flanking makers, AHGS4584 and GM672 can be applied to conduct MAS. The map distance between two markers was 2.55 cM on linkage map with 14.4 megabase (Mb) distance on physical map. Although one major QTL was identified [20], the linkage map distance on entire A01 chromosome was only 34.0 cM constructed by using the S segregating population reported by Qin et al. previously [20] and the QTL region (LOD >3.0) was quite wide, covering approximately one-third of the A01 chromosome. In our current study, the whole linkage group is much larger (157.85 cM) and the identified QTL was located in a much narrow region (2.55 cM), which can be used for MAS with increased confidence. The physical distance was estimated by A genome (*A. duranensis*) sequencing, not directly from the genomes of cultivated peanut, however, the distance was still far. Crossovers could happen between traits (the resistant QTL) and markers, causing recombination, which may lead to certain false positive results and decreased selection accuracy. More closely linked markers should be obtained to increase the efficiency of MAS.

GBS is feasible for large genome species with a low cost [41]. In this study, 12 samples were selected to conduct GBS and the results indicated there were high associations between spotted wilt resistant loci and SNPs on chromosome A01. The SNPs identified at the region should be further validated in order to develop more potential markers, which are closely linked to disease resistance. Besides the disease resistant trait, other traits, for examples, seed maturity, seed size, seed number per pod, seed coat color, and leaf spot resistance, also showed differences in this mapping population. GBS can help to develop SNP markers associated to the traits mentioned above and will be available for constructing a high-density linkage map, QTL analysis, and MAS.

## Conclusions

Expression of spotted wilt disease in peanut is highly variable depending on the disease pressure which varies by location and season. Phenotypic distribution results in this study indicated that disease pressure at NFREC was higher than PSREU. By using immunostrip testing, Florida-EP^TM^ ‘113’ proved to be a good variety to efficiently separate resistant and susceptible genotypes. Since visual rating cannot detect asymptomatic infection, many “resistant” looking plants were actually susceptible genotypes. By screening polymorphic makers between Florida-EP^TM^ ‘113’ and Georgia Valencia, only makers located on A01 chromosome co-segregated with spotted wilt resistance. The GBS method also indicated the strongest association between SNP markers and disease resistant loci on A01 chromosome. Other minor SNPs are worthy of investigating further in the future. The A01 linkage map constructed using the Florida-EP^TM^ ‘113’ derived population had good marker collinearity with the physical map. Two QTLs have been identified on chromosome A01. One QTL was PSREU-specific and another was NFREC-specific. The latter one had higher LOD and PVE values than the former one. Historically, the spotted wilt pressure was lower at PSREU, so QTL regions identified based on phenotypic data at NFREC are more reliable with two flanking markers, AHGS4584 and GM672. This major QTL is most likely contributed by PI 576638, a *hirsuta* botanical-type line, introduced from Mexico. Marker enrichment in the region needs to be conducted to perform fine mapping to refine the QTL region. Next generation sequencing technology could be utilized in the future.

## Additional files

Additional file 1: The number of plot, replication, check at different sites and phenotyping methods in F_3_, F_4_ and F_5_ populations. (DOCX 62 kb) Additional file 2: Literature reference of sources of SSR primers screened. (DOCX 49 kb) Additional file 3: TSWV infection results on F_2:4_ population and parental lines (checks) by visual rating in 2013. a) F_2:4_ population at PSREU; b) checks of F_2:4_ population at PSREU; c) F_2:4_ population at NFREC; d) checks of F_2:4_ population at NFREC. (DOCX 66 kb) Additional file 4: TSWV infection results on F_2:5_ population and parental lines (checks) by visual rating in 2014 at NFREC. a) F_2:5_ population; b) checks of F_2:5_ population. (DOCX 48 kb) Additional file 5: PAGE gel images showing genotypes of 12 samples by using polymorphic SSR markers located on A01, A09 and A10 chromosomes. a) GA110 markers located on A01 chromosome; b) AHGS1910 marker located on A01 chromosome; c) AHGS1647 markers located on A09 chromosome; d) AHGS1390 marker located on A10 chromosome. 1: Georgia Valencia, 2: Florida-EP^TM^ ‘113’, 3 to 7: Susceptible plants, 8–12: resistant plants. (DOCX 763 kb)

## Abbreviations

cM: Centi morgan
GBS: Genotyping by sequencing
GLM: General linear model
ICIM: Inclusive Composite Interval Mapping
LG: Linkage group
LOD: Log-of-odds
MAF: Minor allele frequency
MAS: Marker-assisted selection
Mb: Megabase
NFREC: North Florida Research and Education Center
PAGE: Polyacrylamide gel electrophoresis
PCR: Polymerase chain reaction
PI: Plant introduction
PIC: Polymorphism information content
PSREU: Plant Science Research and Education Unit
PVE: Phenotypic variation explained
PVP: Polyvinylpyrrolidone
QTL: Quantitative trait locus
RIL: Recombinant inbred line
SNP: Single nucleotide polymorphism
SSR: Simple sequence repeat
TSWV: Tomato spotted wilt virus

## References

[1] Savage GP, Keenan JI: The composition and nutritive value of groundnut kernels. In The Groundnut Crop. Springer; 1994:173–213. http://link.springer.com/chapter/10.1007%2F978-94-011-0733-4_6.

[2] Kochert G, Halward T, Branch WD, Simpson CE (1991). RFLP variability in peanut (Arachis hypogaea L.) cultivars and wild species. Theor Appl Genet.

[3] Bertrand PF (1997). Georgia plant disease loss estimates. Univ Ga Coop Ext Pub Pathol.

[4] Culbreath AK, Todd JW, Brown SL (2003). Epidemiology and management of tomato spotted wilt in peanut. Annu Rev Phytopathol.

[5] Pandey MK, Monyo E, Ozias-Akins P, Liang X, Guimarães P, Nigam SN, Upadhyaya HD, Janila P, Zhang X, Guo B (2012). Advances in Arachis genomics for peanut improvement. Biotechnol Adv.

[6] Bertioli DJ, Cannon SB, Froenicke L, Huang G, Farmer AD, Cannon EKS, Liu X, Gao D, Clevenger J, Dash S (2016). The genome sequences of Arachis duranensis and Arachis ipaensis, the diploid ancestors of cultivated peanut. Nat Genet.

[7] Ferguson ME, Burow MD, Schulze SR, Bramel PJ, Paterson AH, Kresovich S, Mitchell S (2004). Microsatellite identification and characterization in peanut (A. hypogaea L.). Theor Appl Genet.

[8] He G, Meng R, Newman M, Gao G, Pittman RN, Prakash CS (2003). Microsatellites as DNA markers in cultivated peanut (Arachis hypogaea L.). BMC Plant Biol.

[9] Gautami B, Foncéka D, Pandey MK, Moretzsohn MC, Sujay V, Qin H, Hong Y, Faye I, Chen X, BhanuPrakash A (2012). An international reference consensus genetic map with 897 marker loci based on 11 mapping populations for tetraploid groundnut (Arachis hypogaea L.). PLoS One.

[10] Nagy ED, Guo Y, Tang S, Bowers JE, Okashah RA, Taylor CA, Zhang D, Khanal S, Heesacker AF, Khalilian N (2012). A high-density genetic map of Arachis duranensis, a diploid ancestor of cultivated peanut. BMC Genomics.

[11] Shirasawa K, Bertioli DJ, Varshney RK, Moretzsohn MC, Leal-Bertioli SCM, Thudi M, Pandey MK, Rami JF, Ka DF, Gowda MVC, Qin H, Guo B, Hong Y, Liang X, Hirakawa H, Tabata S, Isobe S (2013). Integrated consensus map of cultivated peanut and wild relatives reveals structures of the a and b genomes of arachis and divergence of the legume genomes. DNA Res.

[12] Khedikar YP, Gowda MVC, Sarvamangala C, Patgar KV, Upadhyaya HD, Varshney RK (2010). A QTL study on late leaf spot and rust revealed one major QTL for molecular breeding for rust resistance in groundnut (Arachis hypogaea L.). Theor Appl Genet.

[13] Nagy ED, Chu Y, Guo Y, Khanal S, Tang S, Li Y, Dong WB, Timper P, Taylor C, Ozias-Akins P (2010). Recombination is suppressed in an alien introgression in peanut harboring Rma, a dominant root-knot nematode resistance gene. Mol Breed.

[14] Sujay V, Gowda MVC, Pandey MK, Bhat RS, Khedikar YP, Nadaf HL, Gautami B, Sarvamangala C, Lingaraju S, Radhakrishan T, Knapp SJ, Varshney RK (2012). Quantitative trait locus analysis and construction of consensus genetic map for foliar disease resistance based on two recombinant inbred line populations in cultivated groundnut (Arachis hypogaea L.). Mol Breed.

[15] Sarvamangala C, Gowda MVC, Varshney RK (2011). Identification of quantitative trait loci for protein content, oil content and oil quality for groundnut (Arachis hypogaea L.). F Crop Res.

[16] Tillman BL, Gorbet DW (2012). Peanut cultivar UFT113.

[17] Mckinney JL (2013). Influence Of Planting Date, Plant Population, And Cultivar On Management Of Spotted Wilt In Peanut (Arachis Hypogaea L.).

[18] Culbreath AK, Gorbet DW, Martinez-Ochoa N, Holbrook CC, Todd JW, Isleib TG, Tillman B (2005). High Levels of Field Resistance to Tomato spotted wilt virus in Peanut Breeding Lines Derived from hypogaea and hirsuta Botanical Varieties. Peanut Sci.

[19] Culbreath AK, Srinivasan R (2011). Epidemiology of spotted wilt disease of peanut caused by Tomato spotted wilt virus in the southeastern US. Virus Res.

[20] Qin H, Feng S, Chen C, Guo Y, Knapp S, Culbreath A, He G, Wang ML, Zhang X, Holbrook CC, Ozias-Akins P, Guo B (2012). An integrated genetic linkage map of cultivated peanut (Arachis hypogaea L.) constructed from two RIL populations. Theor Appl Genet.

[21] Khera P, Pandey MK, Wang H, Feng S, Qiao L, Culbreath AK, Kale S, Wang J, Holbrook CC, Zhuang W (2016). Mapping Quantitative Trait Loci of Resistance to Tomato Spotted Wilt Virus and Leaf Spots in a Recombinant Inbred Line Population of Peanut (Arachis hypogaea L.) from SunOleic 97R and NC94022. PLoS One.

[22] Branch WD, Branch WD (2001). Registration of “Georgia Valencia”peanut. Crop Sci.

[23] Dellaporta SL, Wood J, Hicks JB (1983). A plant DNA minipreparation: version II. Plant Mol Biol Report.

[24] Hopkins MS, Casa AM, Wang T, Mitchell SE, Dean RE, Kochert GD, Kresovich S (1999). Discovery and characterization of polymorphic simple sequence repeats (SSRs) in peanut. Crop Sci.

[25] Liu Z, Feng S, Pandey MK, Chen X, Culbreath AK, Varshney RK, Guo B (2013). Identification of expressed resistance gene analogs from peanut (Arachis hypogaea L.) expressed sequence tags. J Integr Plant Biol.

[26] Gimenes MA, Hoshino AA, Barbosa AVG, Palmieri DA, Lopes CR (2007). Characterization and transferability of microsatellite markers of the cultivated peanut (Arachis hypogaea). BMC Plant Biol.

[27] Leal-Bertioli SCM, José ACVF, Alves-Freitas DMT, Moretzsohn MC, Guimarães PM, Nielen S, Vidigal BS, Pereira RW, Pike J, Fávero AP, Parniske M, Varshney RK, Bertioli DJ (2009). Identification of candidate genome regions controlling disease resistance in Arachis. BMC Plant Biol.

[28] Moretzsohn MC, Hopkins MS, Mitchell SE, Kresovich S, Valls JFM, Ferreira ME (2004). Genetic diversity of peanut (Arachis hypogaea L.) and its wild relatives based on the analysis of hypervariable regions of the genome. BMC Plant Biol.

[29] Moretzsohn MC, Barbosa AVG, Alves-Freitas DMT, Teixeira C, Leal-Bertioli SCM, Guimarães PM, Pereira RW, Lopes CR, Cavallari MM, Valls JFM, Bertioli DJ, Gimenes MA (2009). A linkage map for the B-genome of Arachis (Fabaceae) and its synteny to the A-genome. BMC Plant Biol.

[30] He G, Meng R, Gao H, Guo B, Gao G, Newman M, Pittman RN, Prakash CS (2005). Simple sequence repeat markers for botanical varieties of cultivated peanut (Arachis hypogaea L.). Euphytica.

[31] Liang X, Chen X, Hong Y, Liu H, Zhou G, Li S, Guo B (2009). Utility of EST-derived SSR in cultivated peanut (Arachis hypogaea L.) and Arachis wild species. BMC Plant Biol.

[32] Bertioli DJ, Moretzsohn MC, Madsen LH, Sandal N, Leal-Bertioli SCM, Guimarães PM, Hougaard BK, Fredslund J, Schauser L, Nielsen AM (2009). An analysis of synteny of Arachis with Lotus and Medicago sheds new light on the structure, stability and evolution of legume genomes. BMC Genomics.

[33] Cuc LM, Mace ES, Crouch JH, Quang VD, Long TD, Varshney RK (2008). Isolation and characterization of novel microsatellite markers and their application for diversity assessment in cultivated groundnut (Arachis hypogaea). BMC Plant Biol.

[34] Peng Z, Gallo M, Tillman BL, Rowland D, Wang J (2016). Molecular marker development from transcript sequences and germplasm evaluation for cultivated peanut (Arachis hypogaea L.). Mol Genet Genomics.

[35] Tang Wang C, Dao Yang X, Xu Chen D, Lin Yu S, Zhen Liu G, Yi Tang Y, Zhi Xu J (2007). Isolation of simple sequence repeats from groundnut. Electron J Biotechnol.

[36] Macedo SE, Moretzsohn MC, Leal-Bertioli SCM, Alves DMT, Gouvea EG, Azevedo VCR, Bertioli DJ (2012). Development and characterization of highly polymorphic long TC repeat microsatellite markers for genetic analysis of peanut. BMC Res Notes.

[37] Moretzsohn MC, Leoi L, Proite K, Guimarães PM, Leal-Bertioli SCM, Gimenes MA, Martins WS, Valls JFM, Grattapaglia D, Bertioli DJ (2005). A microsatellite-based, gene-rich linkage map for the AA genome of Arachis (Fabaceae). Theor Appl Genet.

[38] Wang H, Penmetsa RV, Yuan M, Gong L, Zhao Y, Guo B, Farmer AD, Rosen BD, Gao J, Isobe S, Bertioli DJ, Varshney RK, Cook DR, He G (2012). Development and characterization of BAC-end sequence derived SSRs, and their incorporation into a new higher density genetic map for cultivated peanut (Arachis hypogaea L.). BMC Plant Biol.

[39] Koilkonda P, Sato S, Tabata S, Shirasawa K, Hirakawa H, Sakai H, Sasamoto S, Watanabe A, Wada T, Kishida Y (2012). Large-scale development of expressed sequence tag-derived simple sequence repeat markers and diversity analysis in Arachis spp. Mol Breed.

[40] Fountain J, Qin H, Chen C, Dang P, Wang ML, Guo B (2011). A Note on Development of a Low-cost and High-throughput SSR-based Genotyping Method in Peanut (Arachis hypogaea L.). Peanut Sci.

[41] Elshire RJ, Glaubitz JC, Sun Q, Poland JA, Kawamoto K, Buckler ES, Mitchell SE (2011). A robust, simple genotyping-by-sequencing (GBS) approach for high diversity species. PLoS One.

[42] Glaubitz JC, Casstevens TM, Lu F, Harriman J, Elshire RJ, Sun Q, Buckler ES (2014). TASSEL-GBS: A high capacity genotyping by sequencing analysis pipeline. PLoS One.

[43] Langmead B, Salzberg SL (2012). Fast gapped-read alignment with Bowtie 2. Nat Methods.

[44] Bradbury PJ, Zhang Z, Kroon DE, Casstevens TM, Ramdoss Y, Buckler ES (2007). TASSEL: software for association mapping of complex traits in diverse samples. Bioinformatics.

[45] Yu J, Pressoir G, Briggs WH, Bi IV, Yamasaki M, Doebley JF, McMullen MD, Gaut BS, Nielsen DM, Holland JB (2006). A unified mixed-model method for association mapping that accounts for multiple levels of relatedness. Nat Genet.

[46] Wang J, Li H, Zhang L, Meng L: Users’ manual of QTL IciMapping version 3.2. Quant Genet Group, Inst Crop Sci Chinese Acad Agric Sci (CAAS), Beijing 2012, 100081. http://www.sciencedirect.com/science/article/pii/S2214514115000161.

[47] Van Ooijen JW (2006). JoinMap 4.

[48] Kosambi DD (1943). The estimation of map distances from recombination values. Ann Eugen.

[49] Von Bargen S, Salchert K, Paape M, Piechulla B, Kellmann JW (2001). Interactions between the tomato spotted wilt virus movement protein and plant proteins showing homologies to myosin, kinesin and DnaJ-like chaperones. Plant Physiol Biochem.

[50] Li H, Ye G, Wang J (2007). A modified algorithm for the improvement of composite interval mapping. Genetics.

[51] Mandal B, Pappu HR, Culbreath a K, Pathology P: Factors Affecting Mechanical Transmission of Tomato spotted wilt virus to Peanut (Arachis hypogaea). 2001(December):1259–1263. 10.1094/PDIS.2001.85.12.1259 30831787

[52] Singh BD, Singh AK (2015). Marker-Assisted Plant Breeding: Principles and Practices.

[53] Weinig C, Schmitt J (2004). Environmental effects on the expression of quantitative trait loci and implications for phenotypic evolution. Bioscience.

[54] Collard BCY, Jahufer MZZ, Brouwer JB, Pang ECK (2005). An introduction to markers, quantitative trait loci (QTL) mapping and marker-assisted selection for crop improvement: The basic concepts. Euphytica.

[55] Wang H, Pandey M, Qiao L, Qin H, Culbreath A, He G, Varshney R, Scully B (2013). Genetic mapping of quantitative trait loci analysis for disease resistance Using F2 and F5 Generation-based Genetic Maps Derived from “Tifrunner” × “GT-C20” in Peanut. Plant Genome.

[56] Stevens MR, Scott SJ, Gergerich RC (1991). Inheritance of a gene for resistance to tomato spotted wilt virus (TSWV) from Lycopersicon peruvianum Mill. Euphytica.

[57] Boiteux LS, De Avila AC (1994). Inheritance of a resistance specific to tomato spotted wilt tospovirus in Capsicum chinense “PI 159236.”. Euphytica.

[58] Chen X, Culbreath A, Brenneman T, Holbrook Jr C, Guo B: Identification and cloning of TSWV resistance gene (s) in cultivated peanuts and development of markers for breeding selection. In American Phytopathological Society Abstracts; 2008. https://www.ars.usda.gov/research/publications/publication/?seqNo115=226033.

[59] Chen X, Wang ML, Holbrook C, Culbreath A, Liang X, Brenneman T, Guo B (2011). Identification and characterization of a multigene family encoding germin-like proteins in cultivated peanut (Arachis hypogaea L.). Plant Mol Biol Report.

[60] Barrientos-Priego L, Isleib TG, Pattee HE (2002). Variation in Oil Content Among Mexican and Peruvian hirsuta Peanut Landraces and Virginia-Type hypogaea Lines 1. Peanut Sci.

[61] Isleib TG, Holbrook CC, Gorbet DW (2001). Use of plant introductions in peanut cultivar development. Peanut Sci.

[62] Holbrook CC, Stalker HT (2003). Peanut breeding and genetic resources. Plant Breed Rev.

