# Supplementary material for: Identification of major QTLs underlying tomato spotted wilt virus resistance in peanut cultivar Florida-EPTM ‘113’
Source: BMC Genet. 2016 Sep 6;17(1):128. doi: 10.1186/s12863-016-0435-9 (PMC5012072; doi:10.1186/s12863-016-0435-9)
Supplement: Additional file 1: — The number of plot, replication, check at different sites and phenotyping methods in F3, F4 and F5 populations. (DOCX 62 kb) [file 12863_2016_435_MOESM1_ESM.docx]

Table S1

| Year | Number | Site | |
| --- | --- | --- | --- |
|  |  | PSREU | NFREC |
| 2012 (F_2:3_) | Method | Visual rating | Visual rating  Immunostrip testing |
|  | Line | 163 | 163 |
|  | Line without phenotyping | 26 | 26 |
|  | Check plot | 21 | 21 |
|  | Replication | 1 | 1 |
|  | Total | 210 | 210 |
| 2013 (F_2:4_) | Method | Visual Rating | Visual rating  Immunostrip testing |
|  | Line | 163 | 163 |
|  | Line without phenotyping | 1 | 1 |
|  | Check plot | 28 | 28 |
|  | Replication | 2 | 2 |
|  | Total | 384 | 384 |
| 2014 (F_2:5_) | Method | - | Visual rating |
|  | Line | - | 163 |
|  | Line without phenotyping | - | 1 |
|  | Check plot | - | 28 |
|  | Replication |  | 2 |
|  | Total | - | 384 |
| 2015 (F_6_) | Recombinant Inbred Line | - | 163 |
