# Supplementary material for: Identification of major QTLs underlying tomato spotted wilt virus resistance in peanut cultivar Florida-EPTM ‘113’
Source: BMC Genet. 2016 Sep 6;17(1):128. doi: 10.1186/s12863-016-0435-9 (PMC5012072; doi:10.1186/s12863-016-0435-9)
Supplement: Additional file 2: — Literature reference of sources of SSR primers screened. (DOCX 49 kb) [file 12863_2016_435_MOESM2_ESM.docx]

Table S2.

| Marker reference | Number of markers used |
| --- | --- |
| Hopkins et al., 1999 | 2 |
| Gautami et al., 2012 | 3 |
| Liu et al., 2013 | 7 |
| Gimenes et al., 2007 | 8 |
| Leal-Bertioli et al., 2009 | 8 |
| Moretzsohn et al., 2009 | 9 |
| He et al., 2005 | 17 |
| Moretzsohn et al., 2004 | 17 |
| He et.al, 2006 | 18 |
| Liang et al., 2009 | 20 |
| Proite et al., 2007 | 27 |
| He et al. 2003 | 30 |
| Bertioli et al., 2009 | 48 |
| Qin et al., 2011 | 54 |
| Cuc et al, 2008 | 55 |
| Peng et al., 2016 | 57 |
| Wang et al., 2007 | 57 |
| Macedo et al., 2012 | 65 |
| Ferguson et al., 2004 | 127 |
| Moretzsohn et al., 2005 | 128 |
| Wang et al., 2012 | 156 |
| Nagy et al., 2010 | 284 |
| Koilkonda et al., 2012 | 344 |
| Shirasawa et al., 2013 | 1044 |
| Total | 2585 |
