# Supplementary figures and images for: Identification of major QTLs underlying tomato spotted wilt virus resistance in peanut cultivar Florida-EPTM ‘113’
Source: BMC Genet. 2016 Sep 6;17(1):128. doi: 10.1186/s12863-016-0435-9 (PMC5012072; doi:10.1186/s12863-016-0435-9)

**Figure S3.**

a.
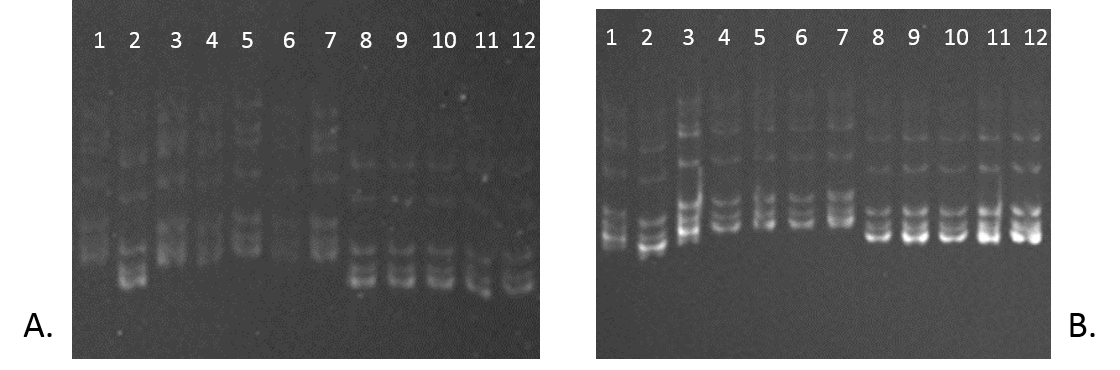
b.

c.
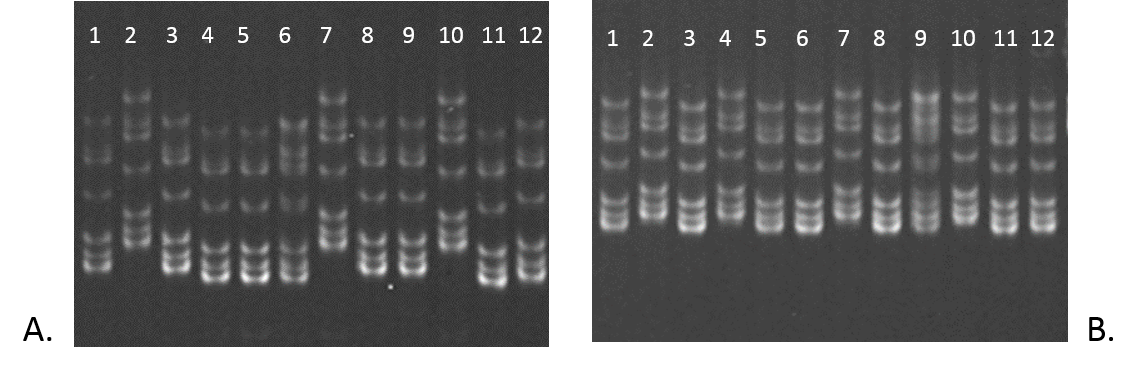
 d.

Supplement: Additional file 5: — PAGE gel images showing genotypes of 12 samples by using polymorphic SSR markers located on A01, A09 and A10 chromosomes. a) GA110 markers located on A01 chromosome; b) AHGS1910 marker located on A01 chromosome; c) AHGS1647 markers located on A09 chromosome; d) AHGS1390 marker located on A10 chromosome. 1: Georgia Valencia, 2: Florida-EPTM ‘113’, 3 to 7: Susceptible plants, 8–12: resistant plants. (DOCX 763 kb) [file 12863_2016_435_MOESM5_ESM.docx]
